# Indication documentation and indication-based prescribing within electronic prescribing systems: a systematic review and narrative synthesis

**DOI:** 10.1136/bmjqs-2022-015452

**Published:** 2023-02-14

**Authors:** Calandra Feather, Nicholas Appelbaum, Ara Darzi, Bryony Dean Franklin

**Affiliations:** 1 Department of Surgery and Cancer, Imperial College London, London, UK; 2 Centre for Medication Safety and Service Quality, Imperial College Healthcare NHS Trust, London, UK; 3 Institute of Global Health Innovation at Imperial College London, London, UK; 4 Department of Practice and Policy, UCL School of Pharmacy, London, UK

**Keywords:** medication safety, decision support, computerised, patient safety

## Abstract

**Background:**

Despite recommendations, documentation of indication on prescriptions and inpatient medication orders is not routinely practised. There has been a recent systematic review of indication documentation for antimicrobials, but not for interventions relating to indication documentation for medication more broadly. Our aims were to 1) identify, describe and synthesise the literature relating to effectiveness of interventions aimed at improving indication documentation and/or indication-based prescribing in both primary and secondary healthcare; 2) synthesise participant perspectives to identify barriers and facilitators to these interventions; and 3) make recommendations for both practice and research.

**Methods:**

A systematic literature search was conducted using Medline, Embase and CINAHL using two search concepts: electronic prescribing systems, and indication documentation and/or indication-based prescribing. Qualitative, quantitative and mixed-methods studies were included; outcome measures and results were extracted to produce a narrative synthesis. Quality appraisal by two independent reviewers was undertaken using the Mixed Methods Appraisal Tool.

**Results:**

We identified 21 studies evaluating interventions to aid indication documentation. Indication documentation was either via free-text, selection from a list, or by use of pre-defined indication-based order sentences for individual medications. For a number of outcomes, there was a mostly positive impact, including appropriateness of the medication order (6 of 8 studies), rates of prescribing error (2/2) and some less commonly reported clinical (2/4) and workflow-related outcomes (2/3). There was a less favourable impact on accuracy of indication documentation and rates of medication use, highlighting some unintended consequences that may occur when implementing new interventions. Participant insights from prescribers and other healthcare professionals complemented quantitative study results, highlighting both facilitators and barriers to indication documentation and the associated interventions. For example, barriers included long drop-down lists and the need to use workarounds to navigate approval systems due to time or knowledge constraints. Facilitating factors included the perceived benefits of indication documentation on communication among the healthcare team and with the patient.

**Conclusion:**

Indication documentation has the potential to improve appropriate prescribing and reduce prescribing errors. However, further benefits to the prescriber, multidisciplinary team and patient may only be realised by developing methods of indication documentation that integrate more efficiently with prescriber workflows.

**PROSPERO registration number:**

CRD42021278495.

**WHAT IS ALREADY KNOWN ON THIS TOPIC**

- Indication documentation on prescriptions and inpatient medication orders is recommended by numerous authorities; however, its practice is not currently routine.

**WHAT THIS STUDY ADDS**

- Interventions to improve indication documentation can increase prescribing appropriateness and reduce prescribing errors; however, accuracy of indication documentation requires further targeted intervention.
- The purpose of indication documentation varies; how this is perceived by the prescriber may influence their motivation to document appropriate and accurate indications.

**HOW THIS STUDY MIGHT AFFECT RESEARCH, PRACTICE OR POLICY**

- This review highlights the need for better methods to document indication in a way that is not burdensome to the prescriber, as well as the need to further evaluate the effect of indication documentation on prescribers and other members of the multidisciplinary team.
- Policy-makers, educators and practice leaders should build on existing successful practice within their own context, promoting indication documentation among prescribers and aiding implementation of routine indication documentation and/or indication-based prescribing.

## Introduction

Medication errors continue to be the leading cause of preventable healthcare-related harm; continued advancement of safer prescribing is therefore required.^1^ Indication documentation is one aspect of prescribing that can aid safer prescribing practices with a potential impact on patients, prescribers and the wider healthcare team.^2–5^ Its purpose is to provide an explicit link between a named medication and its clinical indication,^6^ a practice recommended by various authorities.^7–9^ Despite such recommendations, relatively little progress has been made to incorporate indications into the prescribing workflow.^4^

The advent of electronic prescribing (eP) over the last few decades has seen widespread adoption of eP systems within primary and secondary healthcare. eP offers the opportunity to encourage and facilitate indication documentation at the time of prescribing.^10^ In many eP systems, indication documentation can also be facilitated by selection of indication-based order sentences providing recommended dosing regimens (dose, route, frequency) linked with a particular medication and indication. An indication-based prescribing workflow involves an indication being selected first (rather than a medication) followed by an appropriate medication and dose being suggested to the user. One study evaluating an indication-based prescribing intervention found minimal changes in measured outcomes, with participant interviews identifying contributory factors that may explain this,^11^ highlighting the importance of also studying barriers and facilitators to such interventions.

A recent scoping review of indication documentation for antimicrobials suggests growing awareness of the importance of indication documentation.^12^ Interventions to improve indication documentation generally demonstrated beneficial effects on its prevalence, and almost all studies of prescribing, patient and utilisation outcomes also reported benefits in these areas.^12^

At present, there are no published systematic reviews regarding the use and impact of interventions aiming to improve indication documentation and indication-based prescribing across all medication groups. In addition, there is significant heterogeneity among intervention types and study designs, necessitating careful synthesis. Our aims were therefore to 1) identify, describe and synthesise the literature relating to effectiveness of interventions aimed at improving indication documentation and/or indication-based prescribing in both primary and secondary healthcare; 2) synthesise participant perspectives to identify barriers and facilitators to these interventions and 3) make recommendations for practice and research.

## Methods

### Search strategy

We used two search concepts: ‘eP systems’ and ‘indication documentation/indication-based prescribing’, linked by the Boolean operator ‘AND’. Search terms included relevant synonyms, truncations and spelling alternatives. A test list of nine known papers^11 13–20^ was used to test the search strategy. Searches were conducted on Embase, Medline and CINAHL using relevant subject headings and keywords (online supplemental eTables 1–3) following advice from a subject librarian. Reference lists of included papers were screened for further potentially relevant studies. There were no limits set for date or language.

10.1136/bmjqs-2022-015452.supp1 Supplementary data

### Inclusion/exclusion criteria

Inclusion criteria (online supplemental eTable 4) were that studies had to describe and evaluate interventions and outcomes relating to indication documentation and/or indication-based prescribing. Outcome measures were required to relate to prescribing appropriateness, accuracy, safety, workflow and/or other clinical outcomes. Studies that reported participant insights on a planned or actual intervention were also included. We included both primary and secondary healthcare settings, both ambulatory and inpatient; studies focusing solely on social care settings such as care homes were excluded. Studies were required to have been published as peer-reviewed research papers. We initially included all studies of relevant interventions including those that presented only descriptive data; however, during the synthesis process, studies that did not include effectiveness data were excluded.

### Study selection

The primary reviewer (CF) screened all titles and abstracts and deemed papers either ‘potentially relevant’ or ‘not relevant’ based on the inclusion and exclusion criteria. The second reviewer (BDF) reviewed a random 10%, with any disagreements resolved through discussion until consensus was reached. All ‘potentially relevant’ papers were retrieved for full-text review and a further 10% or 10 full-text papers (whichever was greatest) independently reviewed by the second reviewer. Inter-reviewer agreement was assessed using Cohen’s Kappa.^21^

### Data extraction

Data extracted included author, country, year of publication, study aims and objectives, design, methods, intervention, implementation strategy, setting, population, sample size, duration, eP system, outcome measures, main findings and limitations listed by authors. Qualitative findings were extracted separately and included any relevant participant quotes. Data for two randomly selected papers were extracted independently by the second reviewer to support quality assurance.

### Quality appraisal

The mixed methods appraisal tool (MMAT) was used to assess studies’ methodological quality.^22^ An overall score was calculated for each paper based on scores for each of the five criteria per research method, as per updated MMAT guidance.^23^ Mixed-methods studies were given a score based on the lowest scoring component.^23^ All studies were independently appraised by two reviewers and Cohen’s Kappa calculated for inter-rater reliability; any divergent scores were discussed until a consensus was agreed. Articles were included irrespective of quality score.

### Data synthesis

Due to anticipated heterogeneity of methods and outcome measures, meta-analysis was not considered appropriate. A narrative synthesis was therefore undertaken incorporating both quantitative and qualitative study findings. Participant perspectives were used to identify barriers and facilitators. Guidance from the University of York’s Centre for Reviews and Dissemination^24^ was used as a framework for narrative synthesis, and an overview is provided in online supplemental figure 1. The results of the systematic review combined with information from additional literature^4 12^ were used to create recommendations for practice and research.

10.1136/bmjqs-2022-015452.supp2 Supplementary data

This review follows the Preferred Reporting Items for Systematic Reviews and Meta Analyses (PRISMA) and the Synthesis without meta-analysis (SWiM) reporting guidelines.^25 26^ The protocol was registered prior to commencing data collection on PROSPERO.^27^

## Results

**Figure 1:**
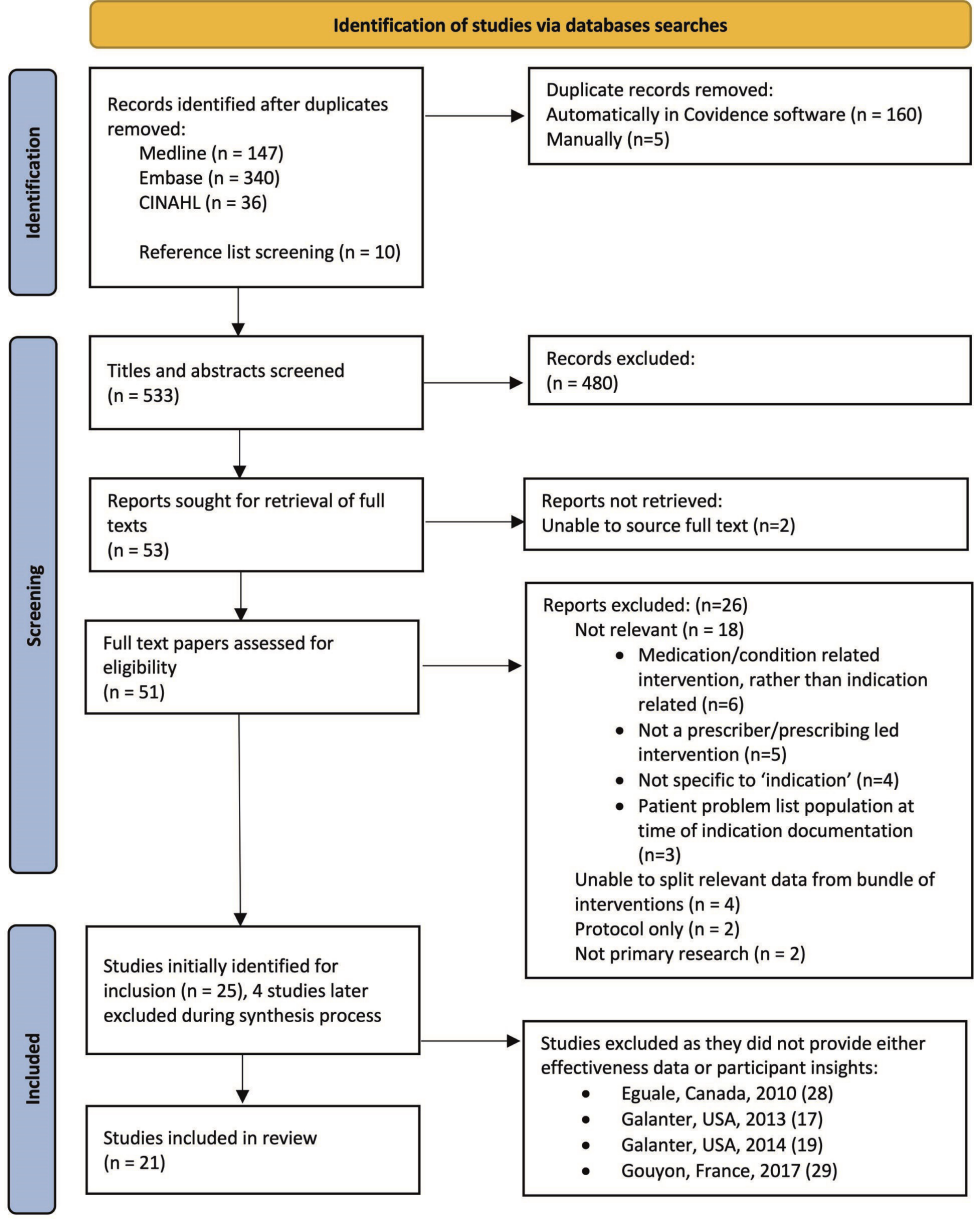
PRISMA flow diagram. Study citation numbers included in brackets for excluded studies. CINAHL, Cumulative Indext to Nursing and allied Health Literature.

After deduplication, a search on 13 September 2021 yielded 523 articles. A further 10 were retrieved from reference lists during full-text screening. Therefore, 533 titles and abstracts were screened, of which 482 were excluded, leaving 51 for full-text screening (figure 1). Following full-text review, 25 articles met the inclusion criteria. The second reviewer screened 55 titles and abstracts with Cohen’s Kappa 0.847 (almost perfect agreement) and 10 full-text articles with Kappa 0.737 (substantial agreement). During synthesis, four further studies were excluded as they did not provide either comparison/effectiveness data or participant perspectives.^17 19 28 29^

### Overview of included studies

The 21 included studies were quantitative (n=15), mixed-methods (n=4) and qualitative (n=2), and included interventions in hospital (both inpatients and outpatients) (n=16), primary care/general practice (n=4) and outpatients only (n=1). The majority focused on adults (n=18); three were in paediatrics. Studies included data from six countries, with the USA (n=14) and Australia (n=5) most common. Studies included participants from the following groups, with eight including more than one group: doctors (n=11), nurses (n=4), pharmacists (n=4), patients/consumers (n=2), advanced practice providers (n=2), certified physician assistants (n=1), ‘prescribers’ (n=5) or not specified (n=4). Publication dates ranged from 2003 to 2021. Table 1 presents an overview of included studies and outcome measures (including effect direction for those with effectiveness data). Online supplemental eTable 5 gives further details including classification of interventions according to the Effective Practice and Organisation of Care taxonomy.^30^

**Table 1:** Summary of studies (with effect direction for those studies with effectiveness data)

First author and year of publication (citation number)	Study design	Intervention description (*Medication type/group targeted in italics*)	Outcome measures, including direction of effect						
			Appropriateness of medication	Accuracy of indication documentation	Rates of medication usage	Error rates	Workflow-related outcomes	Other clinical outcomes	Participant perspectives
**Quantitative randomised controlled trials**									
Meeker, 2016^40^	Cluster RCT	Three separate behavioural interventions to reduce unnecessary antibiotic use (the first two are eP based)—suggested alternatives, accountable justification and peer comparison. *Antimicrobials*	Improved						
Garabedian, 2019^20^	RCT	Indication-based prescribing prototype with patient-specific list of drug choices. *Specific list of medications*				Improved	Improved		
**Quantitative non-randomised studies**									
Herzig, 2015^39^	Interrupted time series	Indication selection for acid-suppressive medication that triggered an alert and guidance to the prescriber to select appropriate indication or to cancel order. *Acid suppressive medications*	Improved		No change				
Vercheval, 2016^31^	Interrupted time series	Policy—mandatory inclusion of indication to start or continue antibiotics and duration or review date (along with bundle of other interventions). *Antimicrobials*			No change			Mixed results	
Richards, 2003^35^	UBA	Web-based antimicrobial approval system, requiring prescriber to select antimicrobial and indication, which then provides the prescriber with an approval number. *Antimicrobials*	Improved		Improved				
Lee, 2008^44^	UBA	Structured insulin order sets, initially on paper then onto eP system. Mandatory for anything but one-time insulin order. *Insulin*						Improved	
Warholak, 2014^43^	UBA	Prescribers asked to provide patient’s diagnosis or indication for use as free text in the notes sections of the e-prescription. *All medications*					Improved		
Metcalfe, 2017^30^	UBA	Approval on antimicrobials via a mandatory indication field. *Antimicrobials*						Improved	
Nomura, 2018^42^	UBA	Incorporation of a provider-selected order indication field with a list of selectable indications for commonly prescribed antimicrobials. Free-text indication documentation could also be used. *Antimicrobials*	Mixed results						
Goss, 2020^18^	UBA	Indication-based prescribing, selection of an antibiotic based on the diagnosis entered, which is then provided as a pre-populated order form. *Antimicrobials*	Improved						
Scardina, 2020^33^	UBA	Addition of indication options (or free-text indication) for ceftriaxone and vancomycin orders. *Antimicrobials*		*			Mixed results		
May, 2021^45^	UBA	Azithromycin order panel with guidance and alternative suggestions. *One antimicrobial medication*	Improved					No change	
Timmons, 2018^38^	Cross-sectional analytical study	The use of drug-specific lists of appropriate indications using institutional guidelines and asked providers to choose an indication at the time of ordering. Or to select ‘other’. *Antimicrobials*	Improved	Decreased					
Stultz, 2019^47^	Cross-sectional analytical study	Use of order sentences for providing meningitis dosing support. *Antimicrobials*				Improved			
**Mixed methods studies**									
Baysari, 2017^11^	CBA and qual interviews	Pre-written orders incorporating authorised indications. *Antimicrobials*	No significant change	No significant change					**√**
Ho, 2020^46^	UBA and quant participant survey	Implementation of a clinical indication library into the prescribing process. *Specific list of medications*						*	**√**
Shemilt, 2019^32^	Quant descriptive and qual survey, focus groups and interviews	Inclusion of indication at time of prescribing for antibiotic therapy and ‘when required’ medications. *Antimicrobials and as required medications*		*					**√**
Beardsley, 2020^36^	Quant descriptive and qual survey	Indication required for antibiotics in three-step process: 1) whether prophylaxis, empirical therapy and definitive therapy; 2) which organ system; 3) which infection. *Antimicrobials*		*					**√**
**Qualitative studies**									
Garada, 2017^37^	Qual interviews	Documenting indication on prescriptions and dispensed medicines labels. *All medication groups*							**√**
Baysari, 2019^34^	Qual interviews	Mandatory indication on eP systems. *All medication groups*							**√**
**Quantitative descriptive studies**									
Gong, 2016^41^	Quant descriptive, participant survey	Behaviour interventions to reduce unnecessary antibiotic use—suggested alternatives, accountable justification (peer comparison and pay-for-performance incentives). *Antimicrobials*							**√**

**√—**Participant perceptions.

*Quantitative descriptive data only (no effectiveness data).

CBA, controlled before-and-after study; eP, electronic prescribing; qual, qualitative; quant, quantitative; RCT, randomised controlled trial; UBA, uncontrolled before-and-after study.

### Quality appraisal

Interrater reliability was initially low at 0.340 (p<0.001); divergent scores were discussed until consensus was met. Of the 21 studies, 12 scored 100%, six 80%, one 60% and two 20% (online supplemental eTable 6). Most common reasons for scoring 80% rather than 100% were for quantitative non-randomised studies for which it was not possible to determine whether confounders were accounted for. Of the four mixed-methods studies, two scored well across both components and therefore scored 100%. The other two scored poorly for one component, giving an overall score of only 20%.

### Intervention types

Interventions to encourage or mandate indication documentation fell into two non-mutually exclusive groups: interventions encouraging indication documentation via selection from a list or free-text entry (n=14),^30–43^ or via use of indication-based order sentences (n=10).^11 18 20 32 40 41 44–47^

#### Indication documentation

Thirteen of the 14 studies were based on either indication selection from a list, or by entering a free-text indication.^30–38 40–43^ In one other study, if a particular ‘inappropriate’ indication was selected for an acid-suppressive medication, the prescriber was presented with guidance on selecting an appropriate indication or cancelling the order.^39^

#### Indication-based order sentences

Interventions based on indication-based order sentences explicitly linked an indication with the medication, along with the dose, frequency, route and so on.^11 18 20 32 40 41 44–47^ When the medication was ordered, the indication was therefore automatically documented. Three of the ten interventions also provided prescribers with suggested alternatives when an indication was entered that was potentially inappropriate for the medication; these may have been for a more appropriate medication choice or for non-pharmacological treatment.^40 41 45^

### Intervention purpose

**Figure 2:**
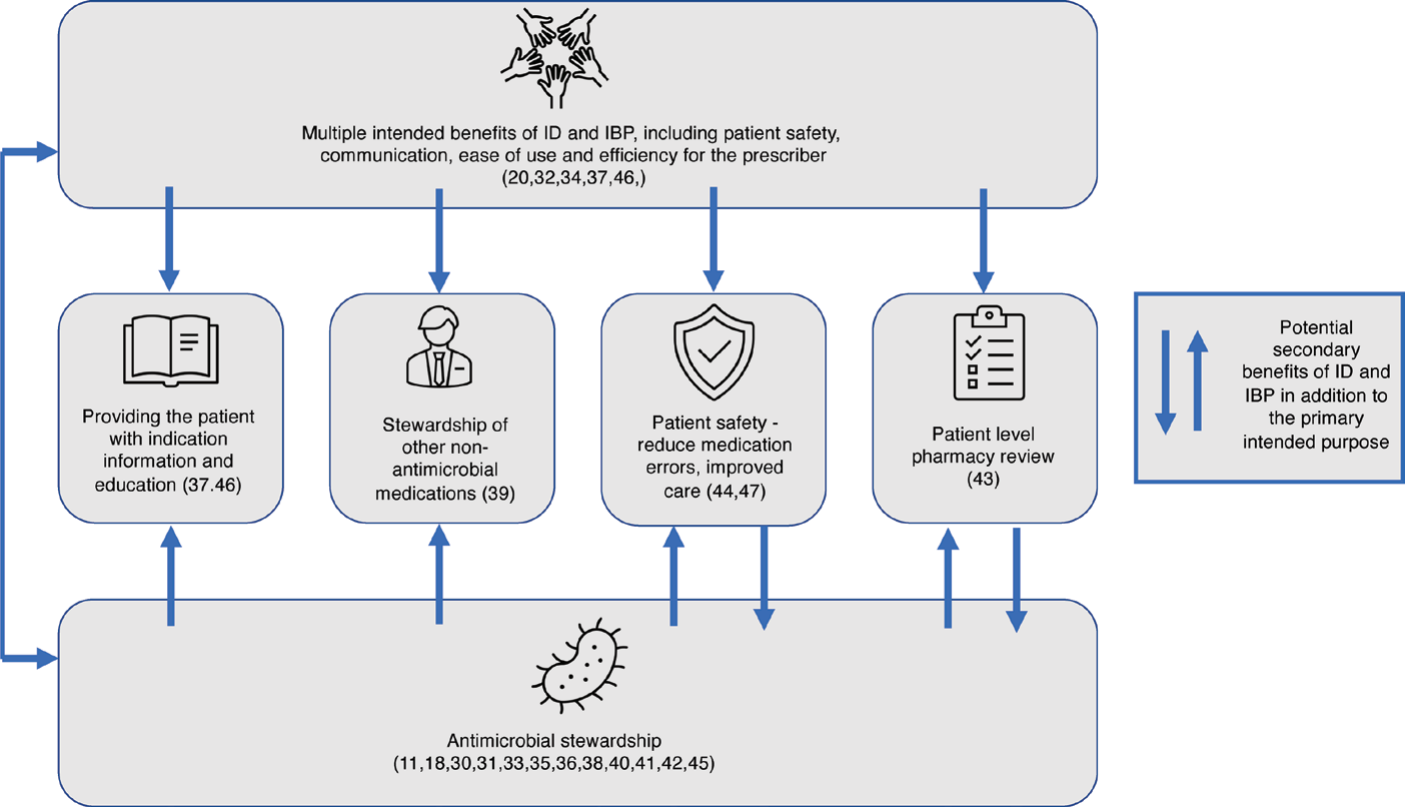
Purpose of indication documentation interventions. Each box indicates the primary intended purpose of the interventions as stated by the individual study authors, the arrows represent the potential secondary benefits as theorised by the authors of this systematic review. IBP, indication-based prescribing; ID, indication documentation. Study references in brackets.

The stated purpose of study interventions is presented in figure 2.

For about half of the studies, interventions were specific to antimicrobials as part of an antimicrobial stewardship programme. The overriding rationale for these interventions was to reduce inappropriate antimicrobial prescribing to reduce resistance at a population level.

The remaining interventions were for the purpose of improving prescribing workflow and/or documentation to improve patient safety, patient information, patient-level review of medication use and to populate patient problem lists within the electronic health record. Some interventions were designed with multiple benefits in mind, such as improving patient safety while also making prescribing easier and more efficient.

### Effectiveness of indication documentation and indication-based prescribing

We identified 15 studies that presented effectiveness data by comparing intervention outcomes against either a pre-intervention period or a parallel control.^11 18 20 30 31 33 35 38–40 42–45 47^ A summary of the study interventions and outcomes is presented in table 1; findings relating to each outcome measure are presented below.

#### Appropriateness of medication

Eight studies assessed the proportion of medication orders deemed to be appropriate, inappropriate or compliant with policy.^11 18 35 38–40 42 45^ Appropriateness was generally defined by study authors as a medication and associated indication that were in accordance with local guidelines or other defined source. Seven studies were in relation to antimicrobials: four for all antimicrobials^11 18 38 42^ and three specifically targeting prescribing for respiratory tract infections.^35 40 45^ The eighth study targeted inappropriate prescribing of acid-suppressive medications as prophylaxis for stress ulcers.^39^

As shown in table 1, overall rates of appropriateness improved in six studies. The intervention associated with greatest improvement was a web-based antimicrobial approval system that required prescribers to select an antimicrobial and indication before being provided with an approval number. This uncontrolled before-and-after study suggested a reduction in the percentage of patients inappropriately treated with ceftriaxone or cefotaxime, from 50% to 27%.^35^ A randomised controlled trial (RCT) reported on multiple interventions relating to indication use (including suggested alternatives, accountable justification and peer comparison) and saw an absolute reduction of 16.0–18.1% in the intervention groups; the control group also experienced an absolute reduction of 11%.^40^ Similarly, an azithromycin order panel with guidance and suggested alternatives was associated with a 12.6% absolute reduction in all inappropriate orders compared with pre-intervention; however, prescriptions with inappropriate dose and duration showed a slight increase.^45^ One intervention advocating use of drug-specific lists of appropriate indications required prescribers to select an indication or to specify ‘other’ and provide a free-text response. The percentage of orders with an appropriate indication was 94.5% for those selected from the list compared with 74.6% that were written as free text.^38^

A number of studies either found no change or mixed results^11 42^; in particular, one study^42^ suggested a decrease in inappropriate orders (p=0.01) post-intervention, but after taking into account orders that had incomplete indication documentation, the difference was no longer statistically significant (p=0.08). Similarly, another study showed no significant change in appropriateness, although sub-analysis suggested a decrease in appropriate prescribing for each additional indication available for a given medication.^11^

#### Accuracy of indication documentation

Effectiveness of interventions on accuracy of indication documentation was reported by two studies.^11 38^ The first used pre-written order sentences with authorised indications, and found no change between control and intervention groups (p=0.1).^11^ Sub-analysis showed that accuracy also decreased for each additional indication available (p=0.0001). The authors felt this was due to incorrect indications being selected or prescribers entering nonsensical text into the free-text indication box; prescribers suggested the latter was a ‘workaround’ to navigate the antimicrobial approval system. The second study compared selection of an indication from a drug-specific list versus free text.^38^ Despite improvements in the appropriateness of medications when selecting an indication from a list, the accuracy of the indication was lower compared with entering free text (OR 0.25; p=0.0043).

#### Rates of medication usage

Medication usage was reported by three studies of interventions aimed at reducing inappropriate prescribing of antimicrobials^31 35^ or acid suppressants.^39^ Two interrupted-time-series studies found overall usage rates to be unaffected by the intervention despite a reduction in medication orders with inappropriate indications,^31 39^ the third study used an uncontrolled before-and-after design and reported a reduction.^35^

#### Prescribing errors

In two studies reporting prescribing error rates, interventions were successful in intercepting and reducing errors.^20 47^ The first used a RCT to compare use of a prototype indication-based prescribing system with two existing eP systems. This required users to start by entering the indication; the prototype then provided drugs of choice. The error rate was significantly lower at 5.5% compared with the average of 29.7% across the two eP systems (p<0.001).^20^ The second was a cross-sectional study that compared error rates for orders with and without an indication-based order sentence (specific to antimicrobials for meningitis). Orders with a meningitis order sentence had an error rate of 19.8% compared with 43.2% for those without (p<0.01).^47^

#### Workflow-related outcomes

One study^33^ demonstrated a reduction in *time to administration* for ceftriaxone, but no change for vancomycin. For the prototype prescribing system using an indication-based prescribing workflow, the *time to complete medication orders* was quicker than with either of the two comparison systems.^20^ A third study^43^ explored use of indication documentation and how this affected incidence and types of drug therapy problems identified by a single clinical pharmacist. Although most *types of problems* identified remained the same, the percentage of prescriptions with *problems requiring pharmacy intervention* reduced from 3.9% of all prescriptions in the pre-intervention phase to 1% post-intervention.

#### Less commonly evaluated clinical outcome measures

The following outcome measures were each reported by one study and are listed as ‘other clinical outcomes’ in table 1. Two studies evaluated use of mandatory indication documentation for antimicrobials; one found *mortality rates* to be unaffected whereas median *length of stay* reduced from 7 to 6 days (p<0.0001).^31^ The second study found that *surveillance rates* increased from 10.5% to 100% and *number of prescriptions without approval* reduced from 179/200 to 0/200.^30^ An uncontrolled before-and-after study found no significant change in *number of patients requiring additional antibiotics* or *number of patients requiring return visit within 30 days* following implementation of an azithromycin order panel with suggested alternatives.^45^ A further uncontrolled before-and-after study measured *glycaemic control rates, percentage of hypoglycaemic/severe hypoglycaemic days* and *risk of hypoglycaemic patient stay*; all improved following introduction of indication-based order sentences for insulin.^44^

### Participant perspectives

Seven studies included participant perspectives on use of indication documentation and indication-based prescribing.^11 32 34 36 37 41 46^

#### Facilitators

Both patient and healthcare participants perceived several mechanisms through which indication documentation and indication-based prescribing could improve clinical practice and could thus facilitate its use. For prescribers and the wider healthcare team, these included facilitating deprescribing, informing prescribers of patient conditions, and increased ability to identify and rectify prescribing errors.^46^ Indication information was also perceived to aid team communication,^34^ particularly at the time of patient transfer between settings.^32^ Indication documentation on outpatient prescriptions and medication labels can also provide patients with information about their medicines and what they are being used for, which was perceived to help patients and their carers.^37 46^

#### Barriers

Practical workflow considerations were a concern for some participants, with long drop-down lists making selection difficult and risking mis-selection.^11 32^ Indication documentation was perceived as time consuming and impractical,^34^ particularly if prescribers were expected to document indications for all medications.^32^ However, participants surveyed by Beardsley *et al* reported that indication documentation was only a ‘minor nuisance’ or ‘occasionally burdensome’ and required only an extra 1–10 or 11–20 seconds, despite the intervention requiring a three-step process.^36^ In contrast, Beardsley *et al* also reported that 21 of 60 participants provided ‘negative [free-text] comments relating to the additional time and/or lack of perceived benefit’. However, this study scored low on MMAT due to insufficient information on its qualitative component. Regarding indication documentation for the purpose of antimicrobial prescribing approval, Baysari *et al* 11 found that junior staff may be pressurised by senior staff to use workarounds to prescribe without approval. In addition, prescribers were found to struggle to define and clarify indications, particularly junior doctors who frequently transcribe inpatient medication orders without necessarily knowing their indication.^34^

Prescribers in two studies from Baysari *et al* felt that inaccuracy of indication documentation may be due to prescriber tendencies to prioritise dose and frequency over indication when selecting from a list, lack of monitoring of selected indications,^11^ and that lack of knowledge and workarounds could lead to poor information quality.^34^ Gong *et al* 41 used a discrete choice experiment to ascertain prescribers’ preferences for interventions to reduce inappropriate antibiotic prescribing following participation in an earlier RCT.^40^ Regardless of the intervention a participant was exposed to, they preferred an intervention that provided suggested alternatives (as indication-based order sentences). However, the earlier trial found peer comparison and justifiable accountability (requiring prescribers to provide justification for the choice of medication by documenting the indication) were more effective.^40^

Lastly, with regard to indication documentation on outpatient prescriptions and medication labels, prescribers and pharmacists were concerned about overcrowded labels and the privacy of patients’ confidential information.^37^ In contrast, patients/consumers largely believed that indication documentation on prescriptions and labels would be beneficial.^37 46^

## Discussion

### Summary of key findings

We identified 21 studies describing interventions to support indication documentation via two mechanisms: indication documentation via selection from a list or as free text, and/or via use of indication-based order sentences. Interventions had diverse purposes, which included improving prescribing workflow, reducing prescribing errors, aiding transfer of information between healthcare professionals, and facilitating patient education.

The most favourable results were for the outcome ‘appropriateness of medication’—although effect sizes varied, six of eight studies showed a positive effect. Other studies demonstrated improvements in prescribing error rates, improved glycaemic control, reduced length of stay, reduced time to complete medication orders and reduced number of prescriptions requiring pharmacy intervention. Participants reported other potential benefits to include facilitating deprescribing, increasing prescribers’ awareness of patients’ conditions and providing medication education for the patient through provision of indication information.

Despite these positive outcomes, it is important to consider some of the less favourable outcomes and unintended consequences of the interventions evaluated. A negative impact was found when evaluating effectiveness of interventions on the accuracy of indication documentation, considered by authors of one study to be due to selection of the indication from a list.^11^ These findings were supported by participant perspectives suggesting that long drop-down lists made selection difficult and risked mis-selection. Other barriers included indication documentation being time consuming and that prescribers prioritised dose and frequency over selection of an accurate indication. The impact of specificity of the indication (eg, urinary tract infection vs pyelonephritis) on accuracy is difficult to assess due to limited information being provided in one of the two studies.^11^

### Comparison with existing literature

Our findings are consistent with Saini *et al*’s scoping review on indication documentation in antimicrobial prescribing.^12^ Our review included fewer studies overall due to more limited inclusion criteria (exclusion of grey literature and indication documentation outside of eP). There were, however, a similar number of studies presenting effectiveness data due to our inclusion of four non-antimicrobial studies. Saini *et al* also provided healthcare worker insights on indication documentation and mapped these as barriers or facilitators using the COM-B behaviour change model (Capacity, Opportunity, Motivation—Behaviour). The results from ours and Saini *et al*’s review appear comparable irrespective of the medication type, suggesting that similar outcomes can be achieved when implementing interventions for medications other than antimicrobials.

Ours and Saini *et al*’s findings relating to participant perspectives resonate with those of Kron *et al*,^4^ whose work was part of a larger project to incorporate indications into the prescribing workflow.^48^ Kron *et al*’s initial work convened multiple stakeholders via online webinars and although it was not published as peer-reviewed research and therefore did not meet our inclusion criteria, it provides in-depth perspectives on indication documentation. To maximise the potential of indication documentation and reduce implementation barriers, Kron *et al* then employed user-centred design principles to develop an indication-based prescribing system that altered the traditional eP workflow. This prototype system allowed users to begin by searching for the indication or selecting a problem from the patient’s existing problem list, and the system then presented the user with a selection of indication-appropriate guideline-based medication options along with order sentences. User-testing results of this prototype were included in our review and demonstrated a reduction in time to prescribe and fewer mouse clicks compared with existing eP systems.^20^ In addition, a further study included in our review^18^ employed an indication-based prescribing workflow for antimicrobials that resulted in an increase in the percentage of appropriate antimicrobial orders. These findings support other authors in the field who propose that an indication-based prescribing workflow has potential to maximise the benefits of indication documentation while limiting the barriers.^2 4 10 48 49^

### Strengths and limitations

Strengths of this review are that, in contrast to Saini *et al*,^12^ we included interventions relating to all medication groups and that the quality of the included studies was appraised independently by two reviewers. While Cohen’s Kappa between the two reviewers was relatively low, this was not unanticipated due to the subjectivity of such appraisal tools. Discussion between the two reviewers allowed for a more thorough appraisal of each paper, often leading to a higher overall score.

This review also has limitations. While every effort was made to conduct a comprehensive search, there is a lack of consistent terminology in this field and therefore our search may not have identified all relevant studies. We only included peer-reviewed research publications; interventions in the grey literature may be missing. The majority of the screening was undertaken by a single reviewer; however, a second reviewer screened and reviewed a proportion of titles and abstracts and then full texts, with almost perfect and substantial agreement at each stage. In addition, data extraction was conducted by a single reviewer; however, a second reviewer extracted data for two randomly selected papers and the original papers were referred back to during the writing-up phase to reduce the likelihood of error. Lastly, publication bias is a possibility, as studies with limited or no effect may be less likely to have been published.

### Recommendations for practice and research

Inclusion of indication documentation at the time of prescribing has potential to benefit the original prescriber, onward prescribers, the wider multidisciplinary team and the patient. Recommendations for practice and research are summarised in box 1.

Box 1 **Recommendations for practice and research relating to indication documentation and indication-based prescribing within electronic prescribing systems**

**Recommendations for practice**

Efforts should be made by quality improvement teams, policy-makers and educators to build on any existing momentum for indication documentation. As indication documentation continues to become commonplace for antimicrobials, this should be capitalised upon as a springboard to extend this practice to further medication groups. Areas of need or high risk should be prioritised.

Raise prescriber awareness of the various purposes of indication documentation to highlight the importance of the accuracy of indication documentation, such as to trigger alerts/reminders or other support mechanisms.

Consideration of the wording used for indication documentation may be required if and when this information may be passed onto patients, such as on discharge documentation, prescription forms or patient-held records.

A myriad of potential barriers and facilitators to successful implementation of indication documentation and indication-based prescribing interventions were identified in this review and elsewhere (4,12,48). Intervention developers and implementers therefore need to work with prescribers and other members of the multidisciplinary team from intervention design through to implementation, to increase the likelihood of success.

**Recommendations for research**

Research into the current methods by which indication and order-sentence libraries are created and maintained by pharmacy informatics teams will allow for a better understanding of the technical challenges in implementing indication documentation and indication-based prescribing interventions.

Further research investigating the impact of indication documentation from the perspective of hospital and community-based clinical pharmacists is required, for example, regarding improved efficiency of deprescribing and pharmacy–prescriber communication.

There was minimal research identified pertaining to the impact of indication documentation and indication-based prescribing on ward-based nurses, even though they check, prepare and administer medications, in addition to communicating medication information to patients. Further research investigating the impact of electronic prescribing-based indication documentation from the perspective of nursing staff is therefore required.

Only two studies included patient participation/feedback (37,46); further research into patients and carers’ perspectives on indication documentation within electronic prescribing systems may be required.

Lastly, effectiveness research conducted in this field should aim to use randomised designs, or at least controlled before-and-after/interrupted-time-series methods to strengthen the evidence; only 5 of 21 studies in the present review employed these stronger designs.

## Conclusion

Indication documentation and indication-based prescribing interventions are being implemented and evaluated across numerous healthcare settings. For some outcomes, studies report a mostly positive impact, particularly for appropriateness of prescribing and prescribing errors. Improvements are required to better integrate indication documentation into prescribing workflows in a way that is acceptable to prescribers and enables accurate indication documentation. In turn, this should facilitate safer prescribing and onward use of indication information to aid communication, decision-making and education for healthcare professionals and patients.

## Data Availability

Data are available on reasonable request.
