# Supplementary material for: Indication documentation and indication-based prescribing within electronic prescribing systems: a systematic review and narrative synthesis
Source: BMJ Qual Saf. 2023 Feb 14;32(6):357–68. doi: 10.1136/bmjqs-2022-015452 (PMC10313979; doi:10.1136/bmjqs-2022-015452)
Supplement: Supplementary data [file bmjqs-2022-015452supp001.pdf]

Online Supplementary documentation

eTable 1 Embase search terms

EMBASE	Subject headings/Mesh	Key terms, including truncation and adjacencies
Concept 1 – CPOE/eP	computerized provider order entry (expanded)<br>decision support system (expanded)<br>physician order entry system	computeri?ed adj2 order entry<br>CPOE<br>electronic* adj1 prescrib*<br>eprescribing<br>e prescribing<br>clinical decision support<br>CDS<br>computeri?ed decision support<br>computer assisted decision making<br>electronic medication management<br>EMM<br>EMMS<br>electronic order entry<br>EPMA<br>physician order entry<br>hospital medication system*<br>medical order entry system*
Concept 2-Indication-based prescribing	drug indication	drug indication<br>indication* based<br>indication* specific<br>indication* for medic*<br>documented adj2 indication*<br>mandatory adj2 indication*<br>prescri* adj2 indication*<br>reason* adj3 drug*<br>reason ajd3 medic*<br>reason adj3 prescri*

eTable 2 Medline search terms

Medline	Subject headings/Mesh	Key terms, including truncation and adjacencies
Concept 1 – CPOE/eP	electronic prescribing<br>decision support systems, clinical<br>medication systems, hospital<br>medical order entry systems	computeri?ed adj2 order entry<br>CPOE<br>electronic* adj1 prescrib*<br>eprescribing<br>e prescribing<br>clinical decision support<br>CDS<br>computeri?ed decision support<br>computer assisted decision making<br>electronic medication management<br>EMM<br>EMMS<br>electronic order entry<br>EPMA<br>physician order entry system*<br>hospital medication system*<br>medical order entry system*
Concept 2-Indication-based prescribing		drug indication<br>indication* based<br>indication* specific<br>indication* for medic*<br>documented adj2 indication*<br>mandatory adj2 indication*<br>prescri* adj2 indication*<br>reason* adj3 drug*<br>reason* ajd3 medic*<br>reason* adj3 prescri*

eTable 3 CINAHL search terms

CINAHL	Subject headings/Mesh	Key terms, including truncation and adjacencies
Concept 1 – CPOE/eP	decision support systems, clinical decision making, computer assisted electronic order entry	computeri?ed N1 “order entry”<br>CPOE<br>eprescribing<br>e prescribing<br>(electronic* N1 prescrib*) or (electronic* N1 prescription*)<br>clinical decision support<br>CDS<br>computeri?ed decision support<br>computer assisted decision making<br>electronic medication management<br>EMM<br>EMMS<br>electronic order entry<br>EPMA<br>physician order entry system*<br>hospital medication system*<br>medical order entry system*
Concept 2- Indication-based prescribing		drug indication<br>indication* based<br>indication* specific<br>indication* for medic*<br>documented N2 indication*<br>mandatory N2 indication*<br>prescri* N2 indication*<br>reason* N3 drug*<br>reason* N3 medic*<br>reason* N3 prescrib*<br>reason* N3 prescription*

eTable 4 Inclusion and exclusion criteria

Criterion	Inclusion	Exclusion
Sources	-Peer reviewed literature from database searches-<br>● Medline<br>● Embase<br>● CINAHL<br>-Reference list screening	Other sources including-<br>● conference abstracts<br>● PhD theses<br>● non-peer reviewed publications
Dates	● No limitation on date	
Study types	● All primary research study designs (relevant systematic reviews were utilised to source other potentially eligible primary research studies by screening the reference list)	● Audits of prescribing that do not relate to the evaluation of an intervention<br>● Protocols without study results
Language	● No language limitations	
Intervention	● Indication-based prescribing using electronic prescribing systems<br>● Indication documentation using electronic prescribing systems<br>● May include data collected regarding a planned intervention that has not yet been implemented<br>● Where the intervention forms part of a larger bundle of components, it was included if it was possible to extract the data relating to indication documentation and/or indication based prescribing	● Studies of paper-based prescribing only<br>● Interventions that required no human-computer interaction at the time of prescribing (e.g., neurolinguistic programming that captured indication information automatically without requiring human verification)
Outcome measures (may including both quantitative and qualitative outcome measures)	● Medication errors<br>● Inappropriate prescribing<br>● Accuracy of indication documentation<br>● Adverse drug events<br>● User perceptions (including pre intervention)<br>● User workflow and team workflow<br>● Staff satisfaction<br>● Efficiency (speed)<br>● Effectiveness (safety)<br>● Other clinical outcomes e.g., mortality rates, length of stay	● Studies without effectiveness data, unless they include participant perceptions via qualitative methods or survey.

Setting	• Primary and secondary healthcare settings, including both clinical and simulation settings.	• Social care settings e.g., studies based solely in care homes
Population- intervention targeting prescribing for-	• General patient populations<br>• Specific patient populations (e.g., renal, paediatrics)<br>• General and specific drug groups	• Studies solely reporting on social care settings such as care home residents
Population- studies assessing interventions targeting prescribers and the wider multi-disciplinary team and patient	• Prescribing healthcare professionals including doctors and non-medical prescribers<br>• Non-prescribing healthcare professionals<br>• Patients and carers/family	

eTable 5 - Summary of studies (Legend – EPOC – Effective Practice and Organisation of Care)

First Author, Date, Country	Intervention grouping	Intervention - brief description	Setting	Study Design	Cochrane EPOC taxonomy classification of intervention and implementation strategies	Main outcome measures	Main results	MMAT quality rating
**Quantitative randomised controlled trials**								
**Meeker, 2016, USA (40)**	Indication documentation +/- use of suggestive alternatives in the form of order sentences	Behaviour interventions to reduce unnecessary antibiotic use - Suggested alternatives, Accountable justification (and peer comparison).	Primary care – multiple primary care clinics	Cluster randomised controlled trial	Health information systems, audit and feedback	Appropriateness - Antibiotic prescribing rates	Mean antibiotic prescribing rates (for anti-biotic inappropriate respiratory tract infections) –<br>Control group- 11% absolute decrease.<br>Suggested alternatives intervention – 16% absolute decrease.<br>Accountable justification intervention – 18.1% absolute decrease.<br>Peer comparison intervention – 16.3% absolute decrease.<br>There was no statistically significant interaction between the interventions.	80%
**Garabedian, 2019, USA (20)**	Indication-based order sentences	Indication-based prescribing prototype with patient-specific list of drug choices.	Prototype for outpatient setting	Randomised controlled trial	Health information systems	Error rates<br><br>Time to complete order<br><br><br><br>System usability scores -	Error rates were 5.5% with the prototype compared with 29.7% with a vendor system.<br>Time to complete a medication order using the prototype was 1.78 minutes, compared with 3.37 minutes with vendor 1 and 2.93 minutes with vendor 2.<br><br>Ease of completing the task was easier with the prototype compared to both vendor 1 and 2.<br>System usability score for the prototype only (nil comparison with vendor 1 and 2) was found to have a mean of 89.7 across all participants.	80%
**Quantitative non-randomised studies**								
**Herzig, 2015, USA (39)**	Indication documentation	Indication selection for acid-suppressive medication (ASM) that triggered an alert and guidance to the prescriber to select appropriate indication or to cancel order.	Secondary care – teaching hospital	Interrupted time series analysis	Health information systems	Appropriateness-<br>The rate of ASM use for ''stress ulcer prophylaxis" outside of ICU (inappropriate prescribing)	There was a reduction in the odds of receiving an inappropriate order to 0.36 at East Campus, and 0.41 at West Campus, plus a change in trend compared to baseline, daily decrease in odds of receiving inappropriate order 1.5% at East campus and 0.9% at West Campus.	100%

First Author, Date, Country	Intervention grouping	Intervention - brief description	Setting	Study Design	Cochrane EPOC taxonomy classification of intervention and implementation strategies	Main outcome measures	Main results	MMAT quality rating
						Rates of ASM use (outside of ICU), overall and at discharge	There was a non-significant reduction in overall rates of use and use on discharge was unchanged.	
**Vercheval, 2016, Belgium (31)**	Indication documentation	Policy - mandatory inclusion of indication to start or continue antibiotics and duration or review date. (along with bundle of other interventions).	Secondary care – teaching hospital	Interrupted time series	Health information systems, educational meetings, educational materials, education outreach visits	Rate of documentation for indication, antibiotic documentation, and duration of therapy<br><br>Occurrence of in-hospital death<br><br>Length of stay<br><br>Compliance with policy-<br>Quality of info recorded by ID physicians (completeness)<br><br>Overall usage of 4 antibiotics	Indication documentation mean percentage increased from 83.4% to 90.3%, average percentage antibiotics documented increased from 87.9% to 95.6%, duration of therapy/review increased from 31.9% to 67.7%.<br><br>Mortality rate remained comparable.<br><br>Length of stay reduced from 7 to 6 days.<br><br>Quality of ID consultation documentation completeness increased from 70.7% to 90.7%.<br><br>The use of the four broad-spectrum antibiotics (meropenem, piperacillin/tazobactam, cefepime, imipenem) was not influenced by the intervention.	100%
**Richards, 2003, Australia (35)**	Indication documentation	Web-based antimicrobials approval system, requiring prescriber to select antimicrobial and indication, which then provides the prescriber with an approval number.	Tertiary care-hospital	Uncontrolled before and after study	Health information systems, educational meetings, tailored intervention - physical removal of cefotaxime and ceftriaxone from certain departments	Gross use of cephalosporins ceftriaxone and cefotaxime (CEFX)<br><br>Gross use of alternative antibiotics<br><br>Compliance with policy – proportion of patients treated empirically with CEFX for an respiratory tract infection without an abnormality on chest xray.	Monthly CEFX use on the wards fell from a mean 38.8DDDs/1000 bed days to 17.6 DDDs/1000 bed days. This was sustained over 15months post intervention period.<br><br>Other broad spectrum antibiotic use remained the same, however gentamicin and benzylpenicillin use increased significantly.<br><br>Proportion of patients treated empirically with CEFX for an respiratory tract infection without an abnormality on chest xray reduced from 50% to 27%.	80%

First Author, Date, Country	Intervention grouping	Intervention - brief description	Setting	Study Design	Cochrane EPOC taxonomy classification of intervention and implementation strategies	Main outcome measures	Main results	MMAT quality rating
**Lee, 2008, USA (44)**	Indication-based order sentences	Structured insulin order sets, initially paper then onto CPOE. Mandatory for anything but one-time insulin order.	Tertiary care-teaching hospital	Uncontrolled before and after study	Health information systems	Glycaemic control rates<br><br>Percentage of hypoglycaemic days<br><br>Percentage of severe hypoglycaemic days and risk of hypoglycaemic patient stay	Regimes including basal insulin improved from 25-29% to 71% across the 3 study periods.<br><br>Percentage of hypoglycaemic days reduced from 3.68% to 2.59%.<br><br>Percentage of severe hypoglycaemic days and relative risk of a hypoglycaemic stay reduced from 0.7% to 0.48%.	60%
**Warholak, 2014, USA (43)**	Indication documentation	Prescribers asked to provide patient's diagnosis or indication for use as free text in the notes sections of the e-prescription.	Primary care – multiple primary care clinics	Uncontrolled before and after study	Health information systems, educational meetings	Incidence and types of potential drug therapy problems identified	The incidence of problems requiring intervention was 3.9% in the pre-implementation phase and reduced to 1% in the post-intervention phase.<br><br>Types of problems requiring pharmacist intervention were-<br>Potential drug–drug interaction, missing information, therapeutic duplication, and excessive dose were the most frequent reasons for interventions in the pre-diagnosis period. Post intervention the most common pharmacist intervention reasons were similar except that excessive dose did not rank among the top three.	100%
**Metcalfe, 2017, Australia (30)**	Indication documentation	Approval on antimicrobials via a mandatory indication field.	Secondary care – teaching hospital	Uncontrolled before and after study	Health information systems, audit and feedback, educational meetings	Surveillance rate<br><br>Rate of approvals<br><br>Compliance with policy – indication documentation	Across the 3 study periods -<br>Surveillance rates – improved from 10.5%, to 65%, to 100%.<br><br>Approval rate improved – number of prescriptions without approval reduced from 179/200, to 70/200 to 0/200.<br><br>Indication documentation improved from 10% to 56.5% to 76.5%.	100%

First Author, Date, Country	Intervention grouping	Intervention - brief description	Setting	Study Design	Cochrane EPOC taxonomy classification of intervention and implementation strategies	Main outcome measures	Main results	MMAT quality rating
**Nomura, 2018, USA (42)**	Indication documentation	Incorporation of a provide-selected order indication field with a list of selectable indications for commonly prescribed antimicrobials. Or free-text indication documentation.	Tertiary care – paediatric teaching hospital	Uncontrolled before and after study	Health information systems and concurrent educational meetings (not specifically related to the eP-based intervention)	Appropriateness – percentage of inappropriate orders when compared with the chart reviewed indication<br><br>Number of inappropriate orders reaching the patient	Inappropriate final orders significantly reduced in the post intervention period from 11.1% to 6.3%. However, when including orders with a an inconsistent or partially inconsistent provider selected indication, there was a non- significant reduction in the number of inappropriate final orders (11.1% to 6.9%).<br><br>A total of 84 inappropriate orders (12%) reached the patient in the pre intervention group and 43 orders (9.3% in the post intervention group (p= 0.15)	80%
**Goss, 2020, USA (18)**	Indication-based order sentences	Indication-based prescribing, selection of an antibiotic based on the diagnosis they enter, which then provided as pre-populated order form	Tertiary care- teaching hospital	Uncontrolled before and after study	Health information systems, educational meeting	Compliance with policy	Selection of a guideline approved antibiotic improved from 67.1% to 72.2%.<br>Minimal improvement noted in selection of appropriate duration of therapy from 24.7% to 31.4%.	80%
**Scardina, 2020, USA (33)**	Indication documentation	Addition of indication options (or free-text indication) for Ceftriaxone and Vancomycin orders.	Tertiary care – paediatric hospital	Uncontrolled before and after study	At time of eP based system under evaluation - Health information systems, educational meetings	Accuracy<br><br>Time to administer antibiotics	Nil pre intervention comparison data for accuracy. In the post-intervention period, indication documentation matched the clinical record 41% of the time for ceftriaxone and 46% for vancomycin.<br><br>The median time to administer ceftriaxone decreased in the post intervention period. There was no significant change in the time to administer vancomycin.	80%
**May, 2021, USA (45)**	Indication-based order sentences	Azithromycin order panel with guidance and alternative suggestions	Primary care clinics	Uncontrolled before and after study	Health information systems	Appropriateness (percentage of inappropriate prescriptions)<br><br>Patients requiring additional antibiotics within 30 days<br><br>Return visits	Overall inappropriate prescriptions of azithromycin reduced by 12.6%, However composite outcomes show a slight increase in prescriptions with inappropriate dose and durations.<br><br>There was no statistically significant change in the number of patients requiring additional antibiotics within 30 days or return visits.	100%

First Author, Date, Country	Intervention grouping	Intervention - brief description	Setting	Study Design	Cochrane EPOC taxonomy classification of intervention and implementation strategies	Main outcome measures	Main results	MMAT quality rating
**Timmons, 2018, USA (38)**	Indication documentation	The use of drug-specific lists of appropriate indications using institutional guidelines and asked providers to choose an indication at the time of ordering. Or to select other.	Secondary care – teaching hospital	Cross-sectional analytic study	Health information systems	Accuracy (indication matching patient diagnosis)<br><br>Appropriateness<br><br>Characteristics of the use of 'other' indication	Matching rates were worse when selecting an indication from a list with a matching percentage of 70.3% compared with 90.4% when selecting 'other' and adding a free text indication.<br><br>Appropriateness was improved with the selection of an indication from a list (94.5%) compared to selecting 'other' 74.6%).<br><br>Prescribers chose 'other' with a free-text indication for 41% of the orders, with a large number being for fluroquinolone orders for respiratory ailments which were not considered appropriate at this institution.	100%
**Stultz, 2019, USA (47)**	Indication-based order sentences	Use of order sentences for providing meningitis dosing support	Tertiary care – paediatric teaching hospital	Cross-sectional analytic study	Health information systems	Dosing error rate<br><br>Other outcomes not relevant to this SR, (regarding sensitivity and specificity of alerts)	There were significantly lower dosing error rates when the antimicrobial was ordered using a meningitis order sentence (19.8%) compared to without (43.2%).	100%
**Mixed methods studies**								
**Baysari, 2017, Australia (16)**	Indication-based order sentences	Pre-written orders incorporating authorised indications	Secondary care – teaching hospital	Controlled before and after study + Qualitative interviews	Health information systems, educational meetings, educational materials	Accuracy of indication<br><br>Appropriateness (national level)<br><br>Compliance with policy (hospital level)<br><br>Participant feedback	No statistically significant change for any primary outcome measures. Sub-analysis showed an increase in negative impact on medications with as the number of possible indications increased.<br><br>Participant feedback - The qualitative interviews *"identified five main factors that contributed to inaccurate documentation of indications in the CPOE, non-compliance to hospital policy and inappropriate antimicrobial use."* The 5 themes are – Dose and frequency took priority over indication; long lists of pre-written orders facilitated errors in selection; lack of monitoring of indications entered into the CPOE system; antimicrobial approval process was time consuming and poorly integrated; pressure	100%

First Author, Date, Country	Intervention grouping	Intervention - brief description	Setting	Study Design	Cochrane EPOC taxonomy classification of intervention and implementation strategies	Main outcome measures	Main results	MMAT quality rating
							from senior doctors to prescribe without obtaining approval.	
**Ho, 2020, USA (46)**	Indication-based order sentences	Implementation of a clinical indication library (CIL) into the prescribing process.	Tertiary hospital	Uncontrolled before and after study + quantitative participant survey and focus group	Health information systems, educational meetings	Operational outcomes – indication documentation prevalence<br><br>Humanistic outcomes-<br>Prescriber and pharmacist views of indication documentation<br><br>Patient views of indication documentation on prescriptions and medicine labels	The proportion of orders with a prepopulated indication increased from 29.8% to 72.3%. After further integration of the intervention into the prescribing workflow, indication documentation for all prescriptions increased to 96%.<br><br>Perceived time spent on indications decreased, understanding of patient profile, conditions improved and better able to reconcile and deprescribe patient medicines. Perceived increased ability to catch wrong medication and dose errors.<br><br>Indications allowed participants to better understand what their medicines were and why it's important to take them and how they worked. It was useful or very helpful to be included on medicines labels.	20%
**Shemilt, 2019, England (32)**	Indication documentation +/- use of indication-based order sentences	Inclusion of indication at time of prescribing for antibiotic therapy and PRN medications.	Secondary care – 2 x district general hospitals, 1 x teaching hospital	Semi-structured interviews and focus groups<br><br>Quantitative descriptive chart review between 3 sites with different prescribing systems	Health information systems	Executive perspectives (chief pharmacists) on the use of clinical indications within the prescription chart design.<br><br>Multidisciplinary team opinions and experiences of indication documentation<br><br>Clarity and accuracy of indication	Triangulation of the chart reviews and qualitative research led to development of 5 themes – clinical workflow, practicality, accuracy, regulation and patient safety.<br><br>Many practical difficulties highlighted including long drop-down lists make selection difficult, impracticality of listing indication for all medications, differences in EPMA systems. However, facilitating factors also described including improved communication between team members, use at time of patient transfer.<br><br>Indication documentation prevalence was highest in hospital A due to use of a mandatory indication field, however	100%

First Author, Date, Country	Intervention grouping	Intervention - brief description	Setting	Study Design	Cochrane EPOC taxonomy classification of intervention and implementation strategies	Main outcome measures	Main results	MMAT quality rating
							accuracy was greater in hospital B for PRN medications which may be due to auto population of indication in an order set.	
**Beardsley, 2020, USA (36)**	Indication documentation	Indication required for antibiotics in three step process. 1st whether prophylaxis, empiric therapy, and definitive therapy. 2nd which organ system, 3rd which infection.	Secondary care – teaching hospital	Quantitative descriptive study with quantitative & free text participant survey	Health information systems	Accuracy<br><br>Correlation of entered indication and final diagnosis for empiric antibiotic orders<br><br>Prescriber perceptions of the requirement to document indication when prescribing antibiotics.	Accuracy of entered indications for all prescriptions was 89%.<br><br>The agreement of the indication documented and the final diagnosis for empiric antibiotic orders was 78.5%.<br><br>Regarding the perceived burden of entering an indication, most participants replied that it required an extra 1-10 or 11-20 seconds and that it was a minor nuisance or occasionally burdensome. 29 of 60 prescribers answered that indication documentation rarely prompted reflection on antibiotic choice. Free-text responses provided suggestions on how to improve the process of indication documentation, with either specific indications to add to the option list, or to have a free-text indication box instead of selection list. 21 gave negative comments relating to the additional time and/or lack of perceived benefit. 6 responses provided support for the intervention.	20%
**Qualitative studies**								
**Garada, 2017, Australia (37)**	Indication documentation	Documenting indication on prescriptions and dispensed medicines labels.	Secondary care – hospital and private	Qualitative interviews	Health information systems	Exploration of participants (prescribers, pharmacist and consumers) views on indication documentation on medication labels, indication wording and potential safety benefits	Key points for each theme-<br>*Potential benefits* – useful, reminder, management in emergency situations, encourage health checks, helps when medicine has multiple indications, helpful for carers.<br>*Describing the indication* – medical terminology may make consumer take condition more seriously, treatment specificity preferred for anti-infectives.	100%

First Author, Date, Country	Intervention grouping	Intervention - brief description	Setting	Study Design	Cochrane EPOC taxonomy classification of intervention and implementation strategies	Main outcome measures	Main results	MMAT quality rating
							Potential safety benefits – reduced confusion with brand names, reduce errors, helps match dose to indication.<br>*Potential limitations*- privacy concerns, overcrowding on the label, prescriber difficulty defining and clarifying indication.	
**Baysari, 2019, Australia (34)**	Indication documentation	Mandatory indication on eP systems.	Secondary care – teaching hospital	Qualitative interviews	Health information systems	Interview questions focused on the current process for indication documentation and gaining approval for antimicrobials	6 Main themes described under 3 headings – Main benefits- Improved communication and prompts prescriber to review medications. Practical difficulties – Not all indications are known and extra time and effort for prescribers. Risks – Workarounds and poor information quality.	100%
**Quantitative descriptive studies**								
**Gong, 2016, USA (41)**	Indication documentation +/- use of suggestive alternatives in the form of order sentences	Behaviour interventions to reduce unnecessary antibiotic use - Suggested alternatives, Accountable justification (peer comparison and pay-for-performance incentives).	Primary care – multiple primary care clinics	Quantitative descriptive participant survey – discreet choice experiment	Health information systems	Discrete choice experience of 5 intervention combinations – Suggested alternatives, accountable justification, peer comparison, pay for performance or additional appointment time.<br>Willingness to pay calculation for each intervention<br><br>Results compared with results from Meeker et al, 2016 (69).	Regardless of the interventions participants were exposed to in the previous study (69), prescribers preferred the suggested alternative intervention, followed by peer comparison and then justifiable accountability.<br><br>Willingness to pay estimated indicated that each intervention would be cheaper that using a pay-for-performance incentive of $200/month.<br><br>Authors concluded that although peer comparison and justifiable accountability were the most effective interventions in the previous trial, stated preferences of prescribers differed and therefore relying only on user feedback may have rules out use of an effective intervention.	100%

eTable 6 – Quality appraisal scores using the Mixed Methods Appraisal Tool, 2018 (22,23) Presented in order of MMAT quality score

First author and year of publication	Study design	Screening Questions		Qualitative studies					Quantitative randomised controlled trials					Quantitative non-randomised studies					Quantitative descriptive studies					Mixed methods studies					Final score
		S1	S2	1.1	1.2	1.3	1.4	1.5	2.1	2.2	2.3	2.4	2.5	3.1	3.2	3.3	3.4	3.5	4.1	4.2	4.3	4.4	4.5	5.1	5.2	5.3	5.4	5.5	
**Baysari, 2019 (34)**	Qual interviews	1	1	1	1	1	1	1																					100%
**Garada, 2017 (37)**	Qual interviews	1	1	1	1	1	1	1																					100%
**Garabedian, 2019 (20)**	RCT	1	1						?	1	1	1	1																80%
**Meeker, 2016 (40)**	Cluster RCT	1	1						1	1	1	?	1																80%
**Herzig, 2015 (39)**	Interrupted time series	1	1											1	1	1	1	1											100%
**Metcalf, 2017 (30)**	UBA	1	1											1	1	1	1	1											100%
**Stultz, 2019 (47)**	Cross-sectional analytic study	1	1											1	1	1	1	1											100%
**Timmons, 2018 (38)**	Cross-sectional analytic study	1	1											1	1	1	1	1											100%
**Warholak, 2014 (43)**	UBA	1	1											1	1	1	1	1											100%
**Vercheval, 2016 (31)**	Interrupted time series	1	1											1	1	1	1	1											100%
**May, 2021 (45)**	UBA	1	1											1	1	1	1	1											100%
**Goss, 2020 (18)**	UBA	1	1											1	1	1	0	1											80%
**Nomura, 2018 (42)**	UBA	1	1											1	1	1	0	1											80%
**Richards, 2003 (35)**	UBA	1	1											?	1	1	1	1											80%
**Scardina, 2020 (33)**	UBA	1	1											1	1	?	1	1											80%
**Lee, 2008 (44)**	UBA	1	1											1	1	1	0	0											60%
**Gong, 2016 (41)**	Quant descriptive, participant survey	1	1																1	1	1	1	1						100%
**Baysari, 2017 (11)**	MM -CBA and qual. interviews	1	1	1	1	1	1	1						1	1	1	1	1						1	1	1	1	1	100%
**Shemilt, 2019 (32)**	MM- Quant descriptive and qual survey	1	1	1	1	1	1	1											1	1	1	1	1	1	1	1	1	1	100%
**Ho, 2020 (46)**	MM, UBA and quant participant survey	1	1											1	1	?	1	1	?	?	1	?	1	1	?	1	1	0	20%
**Beardsley, 2020 (36)**	MM- Quant descriptive and qual. survey	1	1	1	0	0	0	0											1	1	1	?	1	1	0	0	1	0	20%

Table legend, Qual = Qualitative, UBA = Uncontrolled before and after study, Quant = quantitative, RCT = Randomised controlled trial
1 = Yes, 0 = No, ? Cant tell

MMAT Questions for Methodological quality criteria

Screening Questions for all types of study design

S1. Are there clear research questions?

S2. Do the collected data allow to address the research questions?

Questions per Category of study design

1. Qualitative

1.1 Is the qualitative approach appropriate to the research question?
1.2 Are the qualitative data collection methods adequate to answer the research question?
1.3 Are the findings adequately derived from the data?
1.4 Is the interpretation of results sufficiently substantiated by data?
1.5 Is there coherence between qualitative data sources, collection, analysis and interpretation?

2. Quantitative randomised controlled trials

2.1 Is randomization appropriately performed?
2.2 Are the groups comparable at baseline?
2.3 Are there complete outcome data?
2.4 Are outcome assessors blinded to the intervention provided?
2.5 Did the participants adhere to the assigned intervention?

3. Quantitative non-randomised

3.1 Are the participants representative of the target population?
3.2 Are measurements appropriate regarding both the outcome and intervention (or exposure)?
3.3 Are there complete outcome data?
3.4 Are the confounders accounted for in the design and analysis?
3.5 During the study period, is the intervention administered (or exposure occurred) as intended?

4. Quantitative descriptive

4.1 Is the sampling strategy relevant to address the research question?
4.2 Is the sample representative of the target population?
4.3 Are the measurements appropriate?
4.4 Is the risk of nonresponse bias low?
4.5 Is the statistical analysis appropriate to answer the research question?

5. Mixed methods

5.1 Is there an adequate rationale for using a mixed methods design to address the research question?
5.2 Are the different components of the study effectively integrated to answer the research question?
5.3 Are the outputs of the integration of qualitative and quantitative components adequately interpreted?
5.4 Are divergences and inconsistencies between quantitative and qualitative results adequately addressed?
5.5. Do the different components of the study adhere to the quality criteria of each tradition of the methods involved?
