# Supplementary figures and images for: Indication documentation and indication-based prescribing within electronic prescribing systems: a systematic review and narrative synthesis
Source: BMJ Qual Saf. 2023 Feb 14;32(6):357–68. doi: 10.1136/bmjqs-2022-015452 (PMC10313979; doi:10.1136/bmjqs-2022-015452)

Supplementary Figure 1 Synthesis process mapped against the Narrative synthesis framework

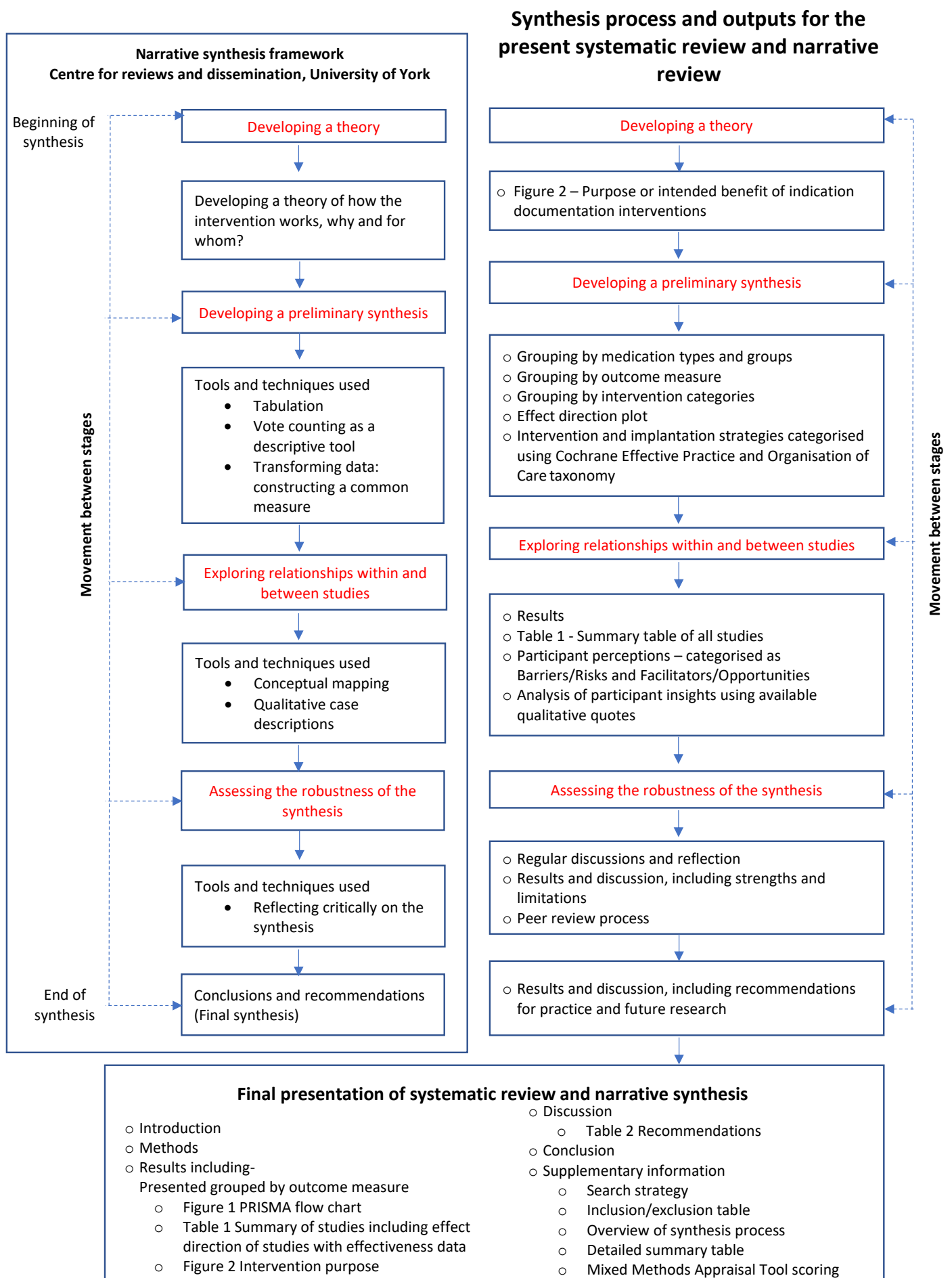

Supplement: Supplementary data [file bmjqs-2022-015452supp002.pdf]
